# The Evolution of the Hallmarks of Aging

**DOI:** 10.3389/fgene.2021.693071

**Published:** 2021-08-26

**Authors:** Maël Lemoine

**Affiliations:** CNRS, ImmunoConcEpT, UMR 5164, Univ. Bordeaux, Bordeaux, France

**Keywords:** aging, geroscience, evolution, unicellular aging, metacellular aging, bilaterians

## Abstract

The evolutionary theory of aging has set the foundations for a comprehensive understanding of aging. The biology of aging has listed and described the “hallmarks of aging,” i.e., cellular and molecular mechanisms involved in human aging. The present paper is the first to infer the order of appearance of the hallmarks of bilaterian and thereby human aging throughout evolution from their presence in progressively narrower clades. Its first result is that all organisms, even non-senescent, have to deal with at least one mechanism of aging – the progressive accumulation of misfolded or unstable proteins. Due to their cumulation, these mechanisms are called “layers of aging.” A difference should be made between the first four layers of *unicellular aging*, present in some unicellular organisms and in all multicellular opisthokonts, that stem and strike “from the inside” of individual cells and span from increasingly abnormal protein folding to deregulated nutrient sensing, and the last four layers of *metacellular aging*, progressively appearing in metazoans, that strike the cells of a multicellular organism “from the outside,” i.e., because of other cells, and span from transcriptional alterations to the disruption of intercellular communication. The evolution of metazoans and eumetazoans probably solved the problem of aging along with the problem of unicellular aging. However, metacellular aging originates in the mechanisms by which the effects of unicellular aging are kept under control – e.g., the exhaustion of stem cells that contribute to replace damaged somatic cells. In bilaterians, additional functions have taken a toll on generally useless potentially limited lifespan to increase the fitness of organisms at the price of a progressively less efficient containment of the damage of unicellular aging. In the end, this picture suggests that geroscience should be more efficient in targeting conditions of metacellular aging rather than unicellular aging itself.

## Introduction

The hallmarks of aging (López-Otín et al., 2013) are mechanisms jointly involved in human aging that are likely to have evolved progressively on top of one another in a multilayered mechanism. To determine how they have evolved is key to understanding how mechanisms of aging interact in humans.

Yet surprisingly, this question has never been investigated. Certainly, the biology of aging (BA) has investigated a variety of pathways with a focus on aging in humans. The Evolutionary Theory of Aging (ETA) has proposed two mechanisms: accumulation of mutations with late onset deleterious effects that decreasing selection pressure cannot eliminate (Medawar, 1952), antagonistic pleiotropy, that is, traits with an early favorable effect and a late deleterious effect (Williams, 1957). These mechanisms have been properly formalized (Hamilton, 1966). Later on, the disposable soma theory, which posits a general mechanism of trade-off between reproduction and repair (Kirkwood, 1977), has laid the foundations for the life history theory of aging. At last, the biodemography of aging (BDA) has investigated the patterns of senescence across the tree of life (Jones et al., 2014; Shefferson et al., 2017). Yet few reviews have sketched which mechanisms of aging are involved in which species (Petralia et al., 2014; Cohen, 2018), and none in an evolutionary perspective.

**FIGURE 1:**
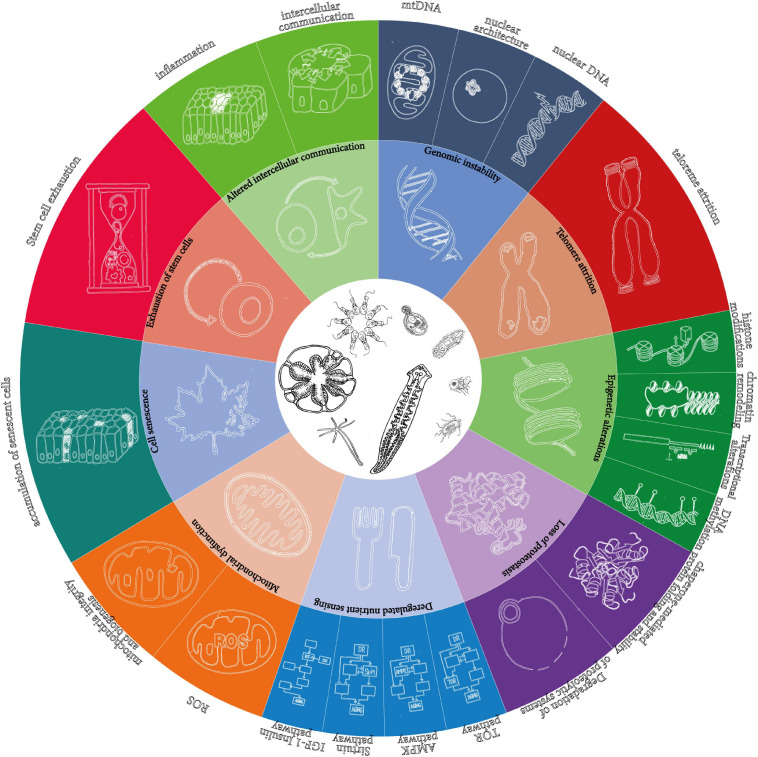
The 9 “synthetic” and the 20 “analytic” hallmarks of aging as originally proposed in López-Otín et al. (2013). The wheel in the original figure proposes only 9 hallmarks of aging, but the text distinguishes more. The present article is based on the detailed hallmarks, which are represented here as an extension of the wheel.

To propose a hypothesis of how human aging has evolved since the Last Universal Common Ancestor (LUCA), the present review relies on 4 working hypotheses:

First, it is based on the list of mechanisms of aging proposed by López-Otín et al. (2013), as the best available, for all its shortcomings (Cohen, 2018). However, instead of keeping the 9 “synthetic” hallmarks, it develops them into the 20 “analytic” hallmarks reviewed in the original paper (Figure 1).

**FIGURE 2:**
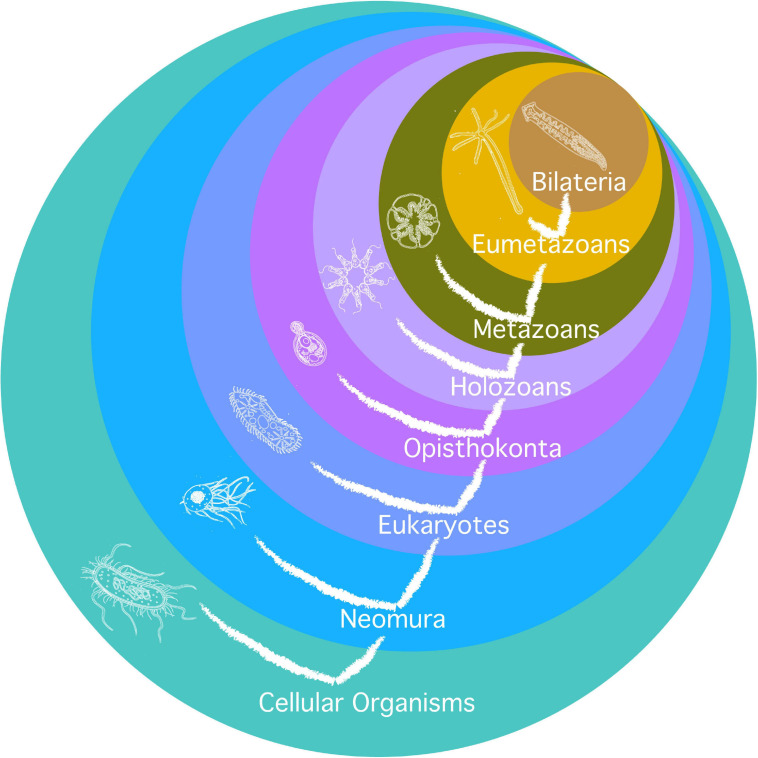
Phylogeny used for the investigation of mechanisms of aging. Circles are clades. Their names are in white. Current species represented in each clade are as remote as possible from humans phylogenetically and hypothetically as close as possible to the early species where the clade originated. The tree of life is also represented on the figure.

Second, it relies on a phylogenetic tree based on (Philippe et al., 2009) and developed for similar purposes in a genealogy of cancer genes (Domazet-Lošo and Tautz, 2010), as illustrated in Figure 2. The limit is that not all major clades are relevant for the investigation of the evolution of aging, and that major transitions in aging may have occurred one after another inside of one such clade (typically, there are several new mechanisms of aging, not just one, from prokaryotes to archaea, from archaea to eukaryotes, and from eumetazoa to bilateria). Additionally, the investigation relies on the investigation of the highest number of species in the clade, or of the most distant species possible in each clade, but it heavily depends on the choice of models in the literature. Typically, the paradigmatic model of eukaryotic aging is *Saccharomyces cerevisiae*, an opisthokont, while less is known on aging in *Chlamydomonas reinhardtii*, a bikont.

Third, this review sets as an ideal experimental evidence that a mechanism is involved in the senescence of a species, but it is often bound to admit it on the basis of likelihood. Indeed, the mere presence in some other species of the components of a mechanism of aging in humans, does not prove that these components are involved in the senescence of this species [as remarked by Cohen (2018)]. Moreover, essential components of a mechanism of aging must have evolved before they participate in a mechanism of aging. Additionally, non-senescent species are less devoid of mechanisms of aging than efficient in countering their actions thanks to mechanisms of anti-aging. In the end, a distinction must be made between a *mechanism of aging* that causes the degradation of a function and is amplifying with time, and an *aging mechanism*, which is less and less functional with time. An example is histones (component), histone modifications (mechanism of aging), and limitation and degradation of the replicative capacity in most eukaryotes and probably neomura (aging mechanism) – against which ciliates have evolved a separation of functions between two nuclei and conjugation. The criterion in such an inquiry is that the action of a mechanism of aging on an aging mechanism is established, e.g., the action of the accumulation of senescent cells in an organ on the progressively degraded function.

Fourth, this review considers that non-senescent species are characterized not by the absence of mechanisms of aging, but the absence of aging mechanisms. What explains this, is the presence of efficient mechanisms of anti-aging that strictly compensate the action of mechanisms of aging. In other terms, the hypothesis is that certain interventions in non-senescent species could in principle make them age. However, a shortened lifespan that follows a gene knock-out in a given species is no evidence that the corresponding protein is involved in a mechanism of anti-aging. An active compensation of the effects of a mechanism of aging must also be established. Typically, the knock-out of genes that code for telomerase leads to a shorter lifespan for yeasts, but this does not mean that yeasts have evolved telomerase to increase their lifespan, although telomerase catalytic subunits are conserved across many branches of the tree of life (Nakamura, 1997).

To establish a hypothesis on the order of appearance of the various layers of bilaterian/human aging in evolution, the present paper investigates the presence and activity of the various mechanisms of aging in each clade from the broadest (cellular organisms), where only one is present, to the narrowest (bilaterians), where all are present. Note that a given mechanism of aging is not necessarily present in all species in the taxon, as it may be lost in some. It must be present both in humans (bilaterians) and in another current species in the same taxon, but not in any other species in the broader clade, to substantiate the hypothesis that the mechanism appeared at that stage in evolution. A final, important note is that the absence of evidence of a mechanism of aging in a species, is no evidence that it is absent in this species, except when the mechanism of aging supposes parts that do not exist in the organism in question (e.g., there cannot be degradation of nuclear architecture or proteolytic systems in prokaryotes). However, the present article being a review, it relies on as few and as strong hypotheses as possible regarding species where a mechanism of aging has not been documented. The burden of evidence relies on those who are tempted to claim that a given mechanism of aging is likely to have appeared earlier than documented in the corresponding clade.

**FIGURE 3:**
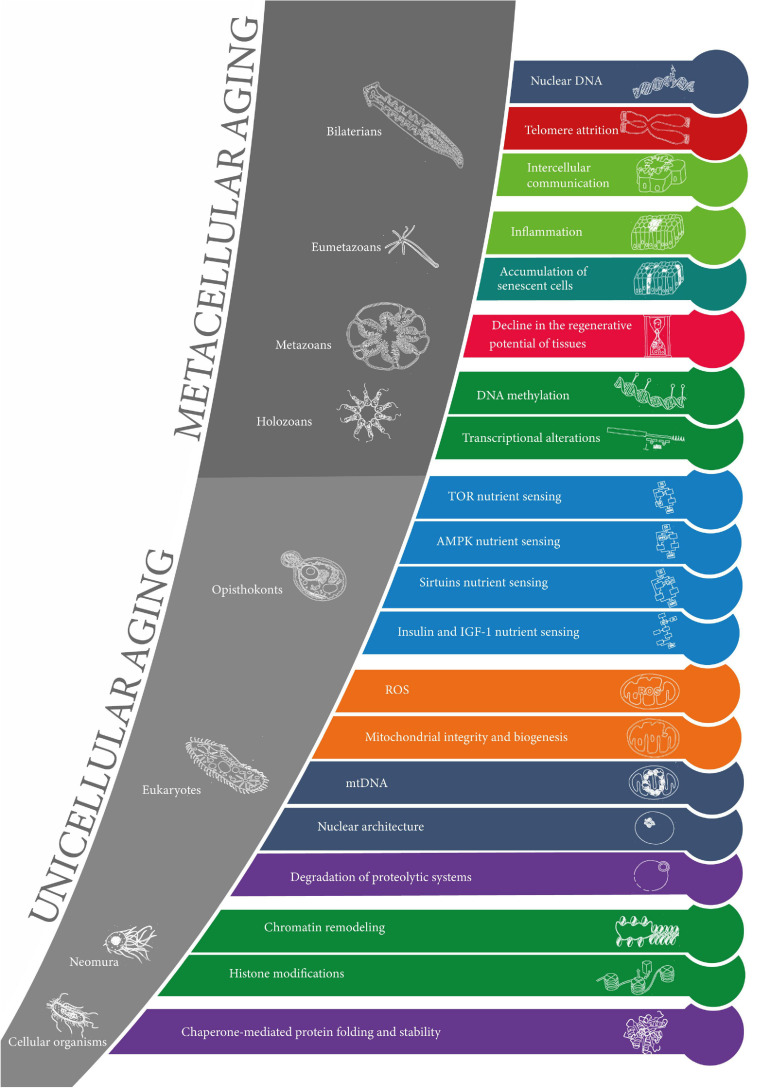
The evolution of the hallmarks of aging. The 20 layers of aging are represented in the order of appearance in evolution from cellular organisms to bilaterians (from the earliest at the bottom to the latest at the top). The colors of the layers correspond to the original 9 “synthetic” hallmarks of aging. Clades are represented in the gray zones along with corresponding model organisms. The sets of layers of unicellular aging and metacellular aging are represented.

The results are summarized in Figure 3.

## Results

### Aging in Cellular Organisms (What Is Common to Bilaterians and Prokaryotes?)

If in any unicellular species, cell division produces two identical cells, and if the species can in principle reproduce indefinitely, then this species cannot be senescent, for daughter cells would be “older at birth” than their mother was so that the species would eventually disappear. Since the end of the 19th century, it has been largely accepted that unicellular organisms divided into identical individuals, and must therefore be non-senescent (Weismann, 1892). However, a distinction is made between chronological lifespan (CLS), i.e., the time individual cells can live before they divide again, and replicative lifespan (RLS), i.e., the number of times individual cells can divide (Ackermann et al., 2007). Individual cells can have a limited CLS and be senescent in that sense, but the difficulty is whether there can be a limit to RLS as well. The discovery that in *S. cerevisiae*, cells divide into unidentical individuals, an aging mother cell and a “rejuvenated” daughter cell, implied that at least some unicellular species display replicative senescence (Mortimer and Johnston, 1959). A further distinction was made between symmetric and asymmetric division (Partridge and Barton, 1993), the former implying non-senescence and characterizing prokaryotes, the latter implying senescence and characterizing eukaryotes (Jazwinski, 1993). When it was established that daughter cells would systematically not have the same lifespan in *Escherichia coli*, a distinction was proposed between ‘morphological asymmetry’ that characterizes division by budding, and ‘functional asymmetry,’ that equally characterized binary fission, at least in *E. coli*, and budding (Stewart et al., 2005). An important consequence is that the evolution of aging does not start with the distinction of germline and soma in multicellular life, as originally speculated (Weismann, 1892; Kirkwood and Cremer, 1982), nor even with eukaryotic life, but as early as in cellular organisms (Ackermann et al., 2007). The most general mechanisms of aging should therefore be investigated in prokaryotes, not eukaryotes. The oldest of the hallmarks of aging cannot therefore be typically eukaryotic, like the degradation of the nuclear architecture or mitochondrial dysfunction.

#### Appearance of the Degradation of Chaperone-Mediated Protein Folding and Stability

It has long been suspected that functional asymmetry, if it existed, should be explained by “the polar localization of cell components” (Stewart et al., 2005) – what has also been called the “senescence factor” in the case of *S. cerevisiae* – a “cytoplasmic factor” (Egilmez and Jazwinski, 1989). Current evidence strongly substantiates the hypothesis that at least part of this senescence factor is aggregated proteins. Indeed, aggregated proteins increase in quantity with the probability of death in a bacterial culture (Maisonneuve et al., 2008a,b). In 9 bacterial cells out of 10, there is only one inclusion body formed by damaged proteins in the dividing cell, which explains how the asymmetric repartition of damage may occur passively, without any dedicated mechanism (Lindner et al., 2008). This discovery weakens the case of a trade-off between repair and reproduction, as repair passively occurs at no energetic cost during cell division, in contrast with the attempt to make functionally asymmetric division a case of the disposable soma theory (Kirkwood, 2005), although there have been attempts to save the theory (Teulière et al., 2020).

Recently, it has also been shown that in situation of antibiotic stress, *E. coli* forms non-viable minicells of up to 20% of the mother cell size, containing damaged proteins (Rang et al., 2018). However, the focus has rather been on the ubiquitous machinery of molecular chaperones. Various classes of chaperones pathways are known to be involved in proteostasis, in bacteria, mostly Hsp70 and GroEL-GroES (Hartl et al., 2011). The various mechanisms molecular chaperones are involved in include holding, unfolding, targeting, pulling and disaggregating misshaped proteins, which are likely to be triggered both in cycles and selectively according to environmental conditions (Mattoo and Goloubinoff, 2014). It has been suggested that increase in the production of aberrant polypeptides in the ribosome, not increase of oxidative damage to proteins nor decline of antioxidation defense, might be responsible for a progressive loss of proteostasis (Nyström, 2003).

In summary, the current picture is that aging in prokaryotes is mainly about the management of abnormal proteins. In most cases, asymmetric division would insure that the lineage does not wear out just because dysfunctional proteins would just aggregate into one inclusion body, and various proteostatic mechanisms involving chaperone molecules play a more minor role in reducing the level of damaged proteins in a lineage (Moger-Reischer and Lennon, 2019).

#### Possibly No Other Hallmarks of Aging in Prokaryotes

To date, loss of proteostasis through “chaperone-mediated protein folding and stability” (López-Otín et al., 2013) is the only mechanism of aging that has been associated with aging in prokaryotes. Other components of mechanisms of aging in narrower taxa are already present. This includes mechanisms of nutrient sensing which is active in starved bacteria under the form of the *s*^*s*^ factor and the alarmone ppGpp. It has been suggested that it might be a factor of aging similar to the *RAS*/cAMP/PKA (in yeast) and the *daf-16* (in *Caenorhabditis elegans*) regulatory pathways (Nyström, 2003, 2004). However, it seems to be involved in CLS only, not in RLS, and in the particular case of “conditional senescence,” that is in circumstances of starvation. Besides, it does not seem to be a case of “*deregulated* nutrient sensing,” but rather, a case of a buffering process with a time limit. Similarly, some repair mechanisms of genetic damage are present, like non-homologous end-joining for double-strand break (Shuman and Glickman, 2007; Lieber, 2010), but they have never been investigated in association with CLS or RLS in bacteria. A possible explanation is that lateral gene transfer (Ochman et al., 2000) may act as a potent replacement strategy in bacteria and discard any form of aging by genomic instability. Epigenetic regulation in prokaryotes does not seem to have been investigated in relation to aging either (Casadesús and Low, 2013).

#### Conclusive Remarks on Aging in Prokaryotes

Our current knowledge seems to suggest that protein aggregation is the oldest and more primitive form of aging – more primitive than DNA damage, and that other mechanisms that will have an anti-aging function later on are already present in bacteria, but do not play that role yet. An important conclusion is also that prokaryotes achieve a form of non-senescence through reproduction by functionally asymmetric division (instead of functionally symmetric division showing that they are not senescent). It has been speculated that in metazoans, in humans in particular, replicative lifespan characterizes stem cells while chronological lifespan characterizes postmitotic cells (Longo et al., 2012). Some human age-related diseases, typically, Alzheimer’s and Parkinson’s, are characterized by progressive decline in proteostasis and abnormal proteins aggregation (Hartl et al., 2011).

### Aging in Neomura (What Is Common to Bilaterians and Archaea?)

A dominant view is that archaea and eukaryotes share a common ancestor and constitute a clade, “neomura,” that branched out of prokaryotes (Cavalier-Smith, 2002; Williams et al., 2013; Cavalier-Smith and Chao, 2020), although alternative hypotheses have been proposed (Forterre, 2013; Mariscal and Doolittle, 2015). Cavalier-Smith proposed that neomura are prokaryotes that have evolved in adaptation to thermophily. Current archaea have further evolved in adaptation to hyperthermophily and hyperacidity. For instance, the evolution of the archaeal cell membrane to less permeability may have contributed to its resistance to chronic energy stress (Valentine, 2007). Archaea and eukaryotes share 19 common traits that sketch what neomura would have looked like (Cavalier-Smith, 2002). None of these traits is characterized in relation to an aging phenotype, and there is currently no study of the involvement of various mechanisms in the senescence of archaea.

A speculation is that moderately challenging conditions (mesophily or thermophily) have put pressure on the evolution of prokaryotic anti-damage mechanisms, making the anti-aging ‘reproductive strategy’ of bacteria insufficient. These anti-damage mechanisms might have then become anti-aging mechanisms in certain conditions.

#### Conservation of the Mechanism of Degradation of Chaperone-Mediated Protein Folding and Stability in Neomura

Archaeal and eukaryotic chaperonins probably synchronize the activity of their rings more efficiently than bacterial chaperonins thanks to a built-in cap (Reissmann et al., 2007). Indeed, eubacterial chaperonins have 7 subunits and a distinct ‘GroES’ Hsp10 cap to close the cylinder while neomura chaperonins would have evolved to 8 or 9 subunits and a built-in cap or lid, which may be preferable for a thermophile (Cavalier-Smith, 2002). Note that hyperthermophiles have lost Hsp70 and Hsp90 that eukaryotes have conserved.

#### Appearance of the Components of ‘Histone Modifications’ and ‘Chromatin Remodeling’ Mechanisms of Aging

The most important, or investigated, evolutions from prokaryotes to archaea regard genomic stability. Based on comparison of eukaryotes, prokaryotes and archaea, it is likely that, from their prokaryote ancestors, neomura have conserved MutL-MutS mismatch repair, transcription coupled repair mechanisms as well as non-homologous end-joining and homologous recombination for double-strand break, and have evolved more sophisticated forms of base excision repair and nucleotide excision repair mechanisms than prokaryotes (White and Allers, 2018). According to Cavalier-Smith, DNA-handling proteins (replicative/repair polymerases and repair and recombination enzymes) have evolved to follow the changes in histones, which may themselves have evolved from H1 linker histones (present in the eubacterial ancestor of neomura) to core histones, both having been later conserved by eukaryotes while archaea lost them (Cavalier-Smith, 2002). An ancestral form of chromatin structure based on tetramer histones may have evolved initially to regulate gene expression and, through the appearance of histones with 8 subunits, have evolved functions of DNA damage prevention in early eukaryotes (Ammar et al., 2012). The earliest function of sirtuins as well may have been chromatin structure regulation, but it may also have been metabolic regulation, as some sirtuins play this role in prokaryotes already (Vaquero, 2009). However, the diversity of proteins involved in the architecture of DNA is puzzling. To reduce the enormous length of unfolded DNA, all kingdoms of life resort to similar strategies of supercoiling, macromolecular crowding, and folding or organization, although structural proteins involved are diverse (Luijsterburg et al., 2008). Even if this remains controversial, there are hints that epigenetics could have emerged at the level of neomura (Blum and Payne, 2019). If this is the case, there is no reason to presuppose that if histone modifications and chromatin remodeling are involved in opisthokontic aging (Yi and Kim, 2020), as there is ample evidence of, it should not be involved in archaeal aging as well to some degree. Indeed, histones and chromatin are highly potent, but vulnerable structures. However, there is no formal evidence of their involvement in archaeal aging at this stage.

In humans, heterochromatin is known to decrease with age and is probably associated to abnormal gene regulation and expression (Greer and Shi, 2012).

### Aging in Eukaryotes (What Is Common to Bilaterians, Bikonts and Amoebozoans?)

There is a huge gap in the evolution of aging from prokaryotes and archaea to eukaryotes, as measured by the important differences between prokaryotes and eukaryotes in general: volume (1,000-fold on average), presence of a nucleus, compartmentalization by endomembrane system and cytoskeleton, presence of mitochondria, regulation of protein function and turnover, and regulation of transcription (Koonin, 2010). While everyone agrees on the endosymbiotic origin of mitochondria, there is disagreement over the origin is a protoeukaryotic cell with an already formed nucleus which later on phagocytized a protobacterion, or a symbiotic relation between an archaeon and a bacterion, the former engulfing the latter and later on evolving a nucleus (O’Malley, 2010). Resolving this dispute will reveal crucial to a finer-grain description of the order of steps in the evolution of aging from archaea to eukaryotes. Plausible preliminary steps of the evolution to eukaryotes that are seemingly not directly related to aging, like the emergence of a cytoskeleton in some archaea (Zaremba-Niedzwiedzka et al., 2017), should provide a basis for speculation about the early evolution of aging.

#### The Appearance of Proteolytic Systems and the Completion of the Mechanism of Loss of Proteostasis

Chaperon-mediated regulation of proteostasis has increased in complexity between prokaryotes and eukaryotes (for a review, see Hartl et al., 2011). Additionally, there is a wide diversity of mechanisms across the domain of eukaryotes: for instance, while *S. cerevisiae*, *Drosophila melanogaster* and *C. elegans* only have one heat shock factor, *C. reinhardtii* (a unicellular green alga) has 2, mammals have 4 and higher plants may have more than 20 (Schmollinger et al., 2013). *Dictyostelium discoideum* presents a very specific regulation of prion-like proteome (Malinovska et al., 2015). In spite of these differences, there is also much in common. For instance, many proteins involved in proteostasis in plastids in plants (e.g., chloroplasts) are conserved from prokaryotes and are involved in senescence (Sakamoto, 2006).

The most important change in the mechanism of loss of proteostasis is the appearance of autophagy. Evidence is now strong that autophagy appeared early with eukaryotic life, as protists that do not present autophagy are likely to simply have lost it (Duszenko et al., 2011). Indeed, in eukaryotes, the turnover of proteins that can also be seen in prokaryotes must be complemented by a turnover of organelles, which would have been the main role of autophagy. Autophagy is triggered in different situations like starvation or infection and modulates cell lifespan (Galluzzi et al., 2008). A possible hypothesis is that it has evolved as a temporary response to nutrient limitation, which also generates ROS and damaged proteins (Pérez-Pérez et al., 2012), and that this mechanism had to adapt later on to a high level of ROS production in mitochondria and/or chloroplasts. Only then, it would have switched from a mechanism of stress resistance to a mechanism of anti-aging.

The recycling of protein aggregates and organelles by autophagy is functionally conserved in eukaryotes, including unicellular alga *C. reinhardtii*, where crATG8, a biomarker of autophagy, is upregulated in the stationary phase of cell culture with a prominent role of the TOR pathway, and is thereby associated with unicellular aging (Pérez-Pérez et al., 2010). In plants too, autophagy is conserved, potentially selective (Li and Vierstra, 2012) and controlled by TOR pathways (Liu and Bassham, 2012), a regulator of growth in changing conditions of nutrient availability (Díaz-Troya et al., 2008). Mutants show accelerated senescence (Doelling et al., 2002). Note, however, that in unicellular algae and plants, there is no documented age-related deregulation of the TOR pathway. The involvement of the degradation of autophagic function in human aging is the subject of intense investigation (Rubinsztein et al., 2011; Cuervo and Wong, 2014).

#### The Conservation of Chromatin Remodeling and Histone Modifications

In ciliates, transcriptionally active macronucleus, strongly involved in clonal aging, contrast with transcriptionally inactive micronucleus, which are not, mainly by histone modifications and chromatin remodeling (Chalker et al., 2013), a contrast that is widely conserved across eukaryotes (Jahn and Klobutcher, 2002; van Wolfswinkel and Ketting, 2010). This constitutes indirect, but strong evidence of their role in aging in the whole clade.

#### Components of the Mechanisms of DNA Methylation and Transcriptional Alterations Appear but Are Not Involved in Aging

DNA methylases, histone-modifying enzymes and RNAi systems seem to have evolved together in eukaryotes (Iyer et al., 2011). Involved in human aging, the machinery of RNA interference [small interfering RNA (siRNA) and micro-RNA (miRNA)] is an original, early evolution of eukaryotes, although it has a functional analog in prokaryotes and is probably not essential to unicellular eukaryotic life, as it has been lost in several branches, e.g., *S. cerevisiae* (Shabalina and Koonin, 2008). RNA interference in prokaryotes seems to essentially consist in an antiviral defense, while it has evolved in a broader regulatory mechanism of transcription in eukaryotes, involving histone modification and DNA methylation (van Wolfswinkel and Ketting, 2010), and not limited to eukaryotes with a multicellular life, as shown by the presence of miRNA and siRNA in *C. reinhardtii* (Molnár et al., 2007). More specific to eukaryotes, the Polycomb Repressive Complex 2 and its analogs in some species, which is involved in transcriptional silencing and remains under the control of RNAi, has probably been selected as a defense against transposons before evolving later on into developmental functions (Shaver et al., 2010). In plants, less than a dozen miRNA genes are widely conserved across species and deeply involved in crucial regulatory networks (development, stress response, and nutritional response), while most others are specific to plants families and have probably no fixed function, suggestive of neutral evolution (Cuperus et al., 2011).

A large part of the regulation of reproduction cycles in many eukaryotes is under the control of such epigenetic mechanisms. There is great variety in these cycles, some showing clear signs of limited RLS. For instance, some ciliates reproduce by endogenous budding, the mother showing signs of functional decline, and late-born daughter cells having more variable lifespan and less progeny; others show alternate phases of differentiation and dedifferentiation and regeneration of adult structures, which was suggested to have evolved to the same abilities in early metazoans (Petralia et al., 2014), although ciliates are bikonts, not opisthokonts. However, a limitation in RLS does not necessarily stems from the epigenetic regulation of the mechanism, which it is not possible to consider an active mechanism of aging at this stage.

#### The Appearance of Mitochondria, Mitochondrial Defects in Biogenesis and ROS Damage

The origin, evolution and multiplicity of functions of mitochondria across eukaryotes is progressively unraveled (Roger et al., 2017). Some eukaryotes (breviates and some ciliates) have no mitochondria, and in others, there are chloroplasts too. It has long been known that a degradation of mitochondria and chloroplasts occurs in stationary phases of culture of unicellular eukaryotes. For instance, in *Tetrahymena pyriformis*, changes in the morphology, number and localization of mitochondria in the stationary phase of cell culture was noted very early (Elliott and Bak, 1964). The unicellular alga *Euglena gracilis* shows the same progression toward stationary phase of the culture and post-mitotic, quiescent state of cells as prokaryotes in the same conditions of limited space and/or resources (Gomez et al., 1974a), which seems to correlate with signs of a loss of proteostasis, and of a progressive degradation of mitochondria (Gomez et al., 1974b). Chloroplasts progressively accumulate photooxidative damage that they partially compensate for by mechanisms of photoprotection, with the effect of photo-inhibition, a progressive diminution of the rate of photosynthesis (Niyogi, 1999).

Endosymbiosis has come with many advantages and as many drawbacks due to poor coordination of cell and mitochondrial genome expression, 1,000:1 ratio of mitochondrial to cell genome in the cell, oxidative damage, potential immune reaction to PAMP and DAMP common to mitochondria and bacteria, and the risk of mutational meltdown that follows from exclusively clonal reproduction of these organelles and is likely not to be balanced by autophagy, chaperone proteins, and regulation of the transcription of the mitochondrial genome (Youle, 2019). Many of these drawbacks are likely to converge in contributing to aging in eukaryotes.

Of note, the role of the accumulation of ROS in aging in general, human aging in particular, has been largely questioned lately (Muller et al., 2007), including in López-Otín et al. (2013), which nevertheless lists ROS as one of the hallmarks of aging. It is widely acknowledged that they are mainly produced by mitochondria, and mainly damage mitochondria. It makes sense to hypothesize their role in aging as early as in eukaryotes. Importantly, there might be strong variation and an increase of ROS damage in narrower clades, in many metazoans in particular, but not in most plants, as shown by the adaptation of the level of production of methionine (Bender et al., 2008).

#### The Appearance of the Degradation of mtDNA and of Nuclear Architecture

Because of the protective properties of histones and of chromatin remodeling, it is likely to take more for DNA damage to become DNA mutations. Moreover, DNA mutations are still much more likely to be a cause of evolution than a cause of aging in unicellular organisms. However, it is to be expected that mtDNA accumulates damage and become a hallmark of aging at this stage, and that the nuclear architecture that protects nuclear DNA will take some of the damage and thus become a hallmark of aging.

In some bacterial species, non-homologous end-joining exists in two forms, canonical and atypical, the former seemingly more frequent in eukaryotes (Bétermier et al., 2014). For instance, being particularly exposed to the genotoxic effects of light, plants and algae have evolved highly efficient repair mechanisms, the imperfection of which is a major cause of their senescence (Bray and West, 2005). Mitochondria and chloroplasts too have basic repair mechanisms that seems to be shared with bacteria, while plants have evolved specific mechanisms in both their mitochondria and chloroplasts (Maréchal and Brisson, 2010). Although evidence is scarce, the degradation of mtDNA is likely to be involved in the aging of all eukaryotic cells. In mammalians in general and in humans in particular, the evidence is more substantial, although the main cause of this degradation has recently been suggested to be less due to ROS production than to faulty replication of mitochondria (Kauppila et al., 2017).

Ciliates illustrate a crucial aspect of the degradation of nuclear architecture. They present nuclear dimorphism – while a micronucleus is inactive during vegetative life and phases of asexual reproduction, one macronucleus (at least) is active in this period. After a certain number of asexual divisions, paramecia age and die if they do not sexually reproduce. A necessary condition of such clonal aging is the progressive degradation of the macronucleus, as a sufficient experimental cause of rejuvenation is macronucleus transplantation (Aufderheide, 1986). The accumulation of damage in the architecture of DNA and of the nucleus, not of individual mutations, is responsible for this, and sexual reproduction by conjugation makes one individual paramecium potentially immortal. Indeed, macronuclei also undergo intense remodeling through fragmentation and *de novo* telomere addition (Jahn and Klobutcher, 2002). Hypotrichous ciliates also age through genomic instability based on a random segregations of genes in their macronuclei, a process not shared among all ciliates (Duerr et al., 2004). In humans, the degradation of nuclear architecture has long been known as the main cause of progeria, traditionally seen as a disease of accelerated aging (Eriksson et al., 2003).

### Aging in Opisthokonts (What Is Common to Bilaterians and Fungi?)

Opisthokonts group animals and fungi. Their ancestor had a unique, posterior flagella (lost later in most organisms, but still present in sperm), and flat mitochondrial cristae (Steenkamp et al., 2006).

#### The Mechanisms of Aging by Loss of Proteostasis Are Conserved

Although major evolutions of proteostasis since prokaryotes are visible in all eukaryotes, they have been mainly studied in opisthokonts. The same form of passive mechanism of anti-aging as in prokaryotes, namely asymmetric repartition of the “senescence factor” during asexual reproduction, can still be seen in *S. cerevisiae*, which reproduces by budding (Zhou et al., 2011), and in *Saccharomyces pombe*, which reproduces by binary fission (Barker and Walmsley, 1999). One part of this senescence factor is oxidatively damaged proteins (Aguilaniu, 2003) and protein aggregates (Liu et al., 2010; Zhou et al., 2011). With increasing RLS, an increased level of Hsp104, the main chaperone in the dissolution of these aggregates, is observed (Xie et al., 2012). However, manipulation (deletion or upregulation) of Hsp104 does not affect lifespan (Kaeberlein et al., 2005; Andersson et al., 2013). Simultaneously, there is a decrease in the level of transcription of genes involved in protein folding and a loss of stoichiometry (Janssens et al., 2015). Although autophagy (reviewed in Reggiori and Klionsky, 2013) is involved in most all classical interventions that successfully extended lifespan in *S. cerevisiae*, it is unclear whether the activation of autophagy in its various forms is sufficient (Tyler and Johnson, 2018).

#### The Mechanisms of Aging Through Histone Modifications, Chromatin Remodeling and Degradation of the Nuclear Architecture Are Conserved

In *S. cerevisiae*, H3 and H2A histone protein levels decrease with increasing replicative aging, and ectopic expression increases it significantly with an effect on longevity (Feser et al., 2010). This is partially under the influence of histone deacetylase Sir2, a member of the sirtuin family of proteins that also participates in several mechanisms of anti-aging (Finkel et al., 2009). H4K16 acetylation also increases with age (Dang et al., 2009). Chromatin remodeling has also been confirmed to be involved in aging (Pegoraro et al., 2009). Misshaped nuclear pore complexes accumulates and the size and shape of the nucleolus changes with age (Pathak et al., 2021).

#### The Conservation of the Mechanisms of Aging Through Mitochondrial Dysfunction

It is unclear how much of the involvement of mitochondrial dysfunction is already present in eukaryotes and not specific to opisthokonts. Longo, Shadel, Kaeberlein, and Kennedy have proposed a network hypothesis of the control of CLS that includes mitochondria, nutrient sensing and stress response in *S. cerevisiae* (Pan et al., 2011; Longo et al., 2012). In addition to the effects of the production of ROS in mitochondria in general, there is a mother-daughter cell gradient of ROS production by mitochondria (Laun et al., 2004). During division, the asymmetric repartition of damaged mitochondria depends on a quality control machinery that involves Mmr1p, a protein localized at the tip of the bud (McFaline-Figueroa et al., 2011). Progressive mitochondrial dysfunction through loss of mtDNA could contribute to age-associated loss of heterozygosity through a dysfunction of iron-sulfur cluster biogenesis (Veatch et al., 2009). In turn, the decline in mitochondrial functioning seems to be caused, at least in part, by a decline in the acidity of vacuoles, which in turn reduces the uptake of neutral amino-acids – but it remains unknown why this would lead to mitochondrial dysfunction (Hughes and Gottschling, 2012).

#### No Mechanism of Aging in Unicellular Eukaryotes Involves Accumulation of Mutations in Nuclear DNA

Although loss of heterozygosity (McMurray, 2003) has been observed in diploid yeast, recent experiments have shown that mutation accumulation is too slow to cause aging in wild type *S. cerevisiae*, either in nuclear DNA or in mitochondrial DNA, and no genome structural arrangement observed at the individual level (Kaya et al., 2015). Only when it is greatly increased beyond a certain threshold in mutant strains, does it have a noticeable effect on lifespan (Lee et al., 2019). That said, extrachromosomal rDNA circles (ERCs) do accumulate in the nucleus of mother cell due to closed mitosis, and are associated to an effect on lifespan countered by Sir2 (Sinclair and Guarente, 1997). However, it has been recently proposed that this just the effect of instability in ribosomal DNA (Kobayashi, 2008; Ganley and Kobayashi, 2014; Hu et al., 2014).

#### The Appearance of Aging Through Deregulated Nutrient Sensing

Opisthokonts have specific biosynthesis and metabolism (Adl et al., 2019), including glycogenesis (Ball et al., 2012). With age, gluconeogenesis and energy storage increase while glycolysis decreases in *S. cerevisiae* (Lin et al., 2001). Autophagy is under the control of nutrient sensing pathways: TOR/Sch9, Ras/cAMP and PKA, AMPK/Snf1 and sirtuins (Sampaio-Marques et al., 2014). First, insulin is a conserved part of a complex hormonal system that regulates growth, phagocytosis, ciliary regeneration, chemotaxis in ciliates (Csaba, 2012). Second, the TOR pathway inhibits autophagy when amino-acid intracellular concentration is high. This pathway becomes hyperactive with age. TOR also seems to increase mitochondrial activity (Pan et al., 2011). Third, the Ras – cAMP – PKA pathway inhibits metabolism and autophagy when glucose in particular is high (Lin et al., 2001). Dietary restriction (DR) has an important effect on lifespan (Fontana et al., 2010), but it remains controversial that it is mediated by sirtuins (Kaeberlein, 2010).

Of note, aging yeast increases in size. Some have proposed the idea that the cell may interpret the consequences of its increasing size as a state of starvation (Yiu et al., 2008) and compensate in a detrimental feed forward loop.

There is overwhelming evidence that the deregulation of nutrient sensing plays a role in human aging, but it is still unclear whether this is a cause or a consequence of other hallmarks of aging.

### Aging in Holozoans (What Is Common to Bilaterians and Choanoflagellates?)

Holozoans evolved toward multicellularity probably to avoid predation and optimize food consumption, and through the evolution of a gene toolkit involving cell adhesion, communication and differentiation (Rokas, 2008). Some choanoflagellates present the interesting case of unicellular organisms capable of a multicellular life (Dayel et al., 2011). In the “colony hypothesis,” the intermediate between unicellular and multicellular organisms was a spheroidal colony with a rudimentary division of labor between external flagellated cells in charge of predation, including perception and locomotion, and internal cells in charge of reproduction, including digestion (Richter and King, 2013). *Salpingoeca rosetta*, a colonial form of choanoflagellates, present 5 different cell types.

There is no well-developed literature on the aging of choanoflagellates specifically. On the one hand, most earlier mechanisms of aging have not been investigated. The TOR pathway is specifically present in choanoflagellates (Shertz et al., 2010), and there is no *a priori* reason why the other mechanisms should not be present.

On the other hand, three hallmarks are both absent from the previous clade of opisthokonts and the next clade of metazoans: DNA methylation, transcriptional alterations, and decline in the regenerative potential of tissues. It can reasonably be hypothesized that the former two appeared in holozoans, while the latter appeared only in metazoans. If this hypothesis is true, holozoans should present ‘conditional senescence’, that is, age through these mechanisms at least in some specific environmental conditions.

First, DNA methylation may have evolved from a mechanism of cell defense in prokaryotes to a mechanism of adaptation in eukaryotes, to a mechanism of intercellular coordination in holozoans and beyond. Any uncoordinated pattern of methylation in holozoans may cause the degradation of the multicellular entity. However, there is no evidence if its involvement in aging. Second, the same is true of the epigenetic machinery of miRNA, which has been lost by *S. cerevisiae*, but is present in other unicellular opisthokonts (Bråte et al., 2018): a plausible hypothesis, which is not verified at this stage, is that it may be involved in aging in multicellular holozoans. Indeed, miRNAs control the expression of genes in time and consequently display a complex pattern of up- and down-regulation, which is also specific to cell types (Smith-Vikos and Slack, 2012).

Cell senescence might also have appeared in multicellular holozoans. It is attested in eumetazoans but has not been investigated in metazoans nor in choanoflagellates. Indeed, programmed cell death (PCD) appeared early in evolution, probably before multicellular life and potentially involving more than 30 different genes (Nedelcu, 2009). It obviously became a vital program for survival in multicellular organisms, although it may have initially evolved as a form of antagonistic pleiotropy (Nedelcu et al., 2011). In particular, sequences coding for *p53* are already present in choanoflagellates and could have evolved for purposes of proliferation control (Pearson and Alvarado, 2008), although there is no experimental evidence of such functional pathway yet (Nedelcu and Tan, 2007). Some processes close to what is observed in apoptosis have been observed in unicellular organisms (Nedelcu, 2009). The hypothesis is therefore that active cell death has evolved first either as an altruistic behavior or as an effect of antagonistic pleiotropy in unicellular organisms (Ameisen, 2002; Nedelcu et al., 2011), and that it became adaptive in multicellular organisms. In the case of an altruistic behavior, it would be consistent to hypothesize that PCD has evolved as an adaptive reaction to some the detrimental effects of some form of cell “senescence,” be it simply the state of aged individual cells. In the case of antagonistic pleiotropy, senescence could have evolved as some sort of resistance to PCD. However, this is too speculative to hypothesize that cell senescence is involved in aging in holozoans.

DNA methylation is not far from becoming the standard biological measurement of chronological aging in humans (Horvath, 2013), although it is still unknown whether the various signatures that have recently been proposed are direct causes of aging, or markers of the activity of maintenance systems, or anything else. Transcriptional alterations associated with shifts in miRNA expressions have been studied mainly in cancer (Iorio et al., 2005), but also in other age-related diseases (Cogswell et al., 2008; Rayner et al., 2010) and, to a lesser extent, in aging more generally (Smith-Vikos and Slack, 2012; Smith-Vikos et al., 2016; Olivieri et al., 2017).

### Aging in Metazoa (What Is Common to Bilaterians and Porifera?)

The extremely long lifespan of sponges (*Porifera*), up to 11,000 years in documented cases (Jochum et al., 2012), associated with no evidence of decline, has led to the claim that they belong to non-senescent species. Sponges have also been proposed as an illustration of the 6 “hallmarks of animal multicellularity,” namely regulated cell cycle, programmed cell death, cell-cell and cell-matrix adhesion, developmental signaling and gene regulation, allorecognition and innate immunity and specialization of cell types (Srivastava et al., 2010). They present the same structure as colonial choanoflagellates with a genetic development program. Most of their cells are called choanocytes, a short-lived cell type organized in epithelia that form channels through which they create a flow by the movement of their flagella (Leys and Hill, 2012). In total, *porifera* present 5–10 different cell types.

To clarify this stage of the evolution of aging, I propose a distinction between *unicellular* aging and *metacellular* aging. Indeed, the engine of the process of aging in bilaterians may be the aging of the cells themselves. But it is important not to simplistically scale up cellular aging to whole organisms (Cohen, 2018). Non-senescent metazoans seem to be able to keep aging at bay thanks to a high proportion of stem cells with a high rate of activity. In other terms, they themselves do not age while their cells do age individually and intrinsically. Senescent metazoans age both because their cells do individually (*unicellular aging*) and because of an organization of multicellular life that leads to an insufficient rate of renewal, something that may be called *metacellular aging*. The default mode of multicellular life seems to have been non-senescence.

#### The Likely Conservation of Unicellular Mechanisms of Aging Through Loss of Proteostasis, Epigenetic Alterations, Genomic Instability, Deregulated Nutrient Sensing, Mitochondrial Dysfunction

The hallmarks of unicellular aging are not well known in Porifera. Protein folding and repair by HSP70 has been studied in Porifera, but mostly in stress and not in unicellular aging (Vallmann et al., 2016). Regarding autophagy, there are only studies of the conservation of proteins involved, like Atg8 (Shpilka et al., 2011). There is no study of the deregulation of nutrient sensing in porifera. Importantly, the genome of metazoan mitochondria is 4 times smaller and contains 1.5 times less genes than the genome of holozoan mitochondria (Lavrov et al., 2005). This compaction is accompanied in particular by codon reassignment of AUA from isoleucine to methionine. There seems to be a correlation between high aerobic metabolic rate and higher levels of methionine (an antioxidant) in mitochondria than in the rest of the cell, which is the case in most animals, and, inversely between lower aerobic metabolic rate and lower levels of methionine, which is the case in Porifera, cnidarians and platyhelminthes (Bender et al., 2008).

There are no studies on the role of histones, chromatin and nuclear architecture in the mechanisms of aging in Porifera, but some molecules they produce have been studied for their pharmacological potential in modulating chromatin remodeling (Luparello et al., 2020). miRNAs have not been studied in relation to aging, but their regulatory role in Porifera has been established (Wheeler et al., 2009).

#### Appearance of the Decline in the Regenerative Potential of Tissues

In Porifera, a system of stem cells is composed of two kinds of cells, archaeocytes, which act both as oocytes and as totipotent stem cells, and choanocytes, which can produce sperm, have physiological functions and act as pluripotent stem cells (Funayama, 2010, 2018; Leys and Hill, 2012). Even dissociated from the organism, they can regrow functional tissue (Custodio et al., 1998). It has long been assumed that stem cells in Porifera have a limitless capacity to regenerate the whole body of the animal (Schröder et al., 2003), notably under the influence of a high basal, normal rate of telomerase production (Koziol et al., 1998). However, it is not limitless, but it takes time and resources that may be better allocated in other functions like growth, sexual reproduction, competition, immunity – so that regeneration in ecological conditions depends on both intrinsic and extrinsic conditions, the availability of a sufficient number of totipotent stem cells playing a crucial role in the process (Henry and Hart, 2005). In Porifera, aging in the wild is likely to be involved when trade-offs are not favorable to regeneration. For that reason, it can be hypothesized that if Porifera undergo conditional senescence, it may be through the exhaustion of stem cells in a protracted state of demand on regeneration that they cannot meet.

It is also likely that the rate of activity of stem cells is regulated through intercellular communication in early metazoans. Intercellular signaling pathways have been particularly studied in Porifera development and involve Wnt, TGF-b, Hedgehog, tyrosine kinase, nuclear receptor, Notch, Jak/STAT (Barolo and Posakoni, 2002). Probably because Porifera are considered non-senescent, the few studies of abnormal signaling in this species do not evoke aging, but rather cancer – for instance, exposition to ectopic Wnt produces abnormal growth (just as they do in cnidarians) and dysfunctional canals (reviewed in Leys and Hill, 2012). Tyrosine kinases in particular are signaling proteins involved in many processes, evolved differently in many forms in different branches (Richter and King, 2013). There are claims that tyrosine kinases are involved in aging in general, but they remain vague (e.g., Hunter, 2009). There is no sign of cell senescence either, although *p53*-driven apoptosis is still present just as it is in choanoflagellates (Rutkowski et al., 2010).

In humans, the exhaustion of stem cells is actively studied as a mechanism both of aging and of cancer (Sharpless and DePinho, 2007; Liu et al., 2019).

### Aging in Eumetazoa (What Is Common to Bilaterians and Cnidarians?)

Many, but not all cnidarians, are non-senescent or at least long-lived species (Petralia et al., 2014). In particular, *Hydra* is characterized by a body almost entirely composed of stem cells of three sorts and a rapid turn-over of cells: all the cells of the body of *Hydra* are replaced within 30 days, this form of maintenance by full renewal probably being the main reason why some of them are non-senescent (Schaible et al., 2015), while others are not, due to imperfect turnover (Schaible et al., 2014).

#### The Conservation of Unicellular Mechanisms of Aging in Early Eumetazoans: Degradation of Proteolytic Systems, ROS Damage, Degradation of the Nuclear Architecture

How cnidarian cells manage protein aggregates does not seem to have been investigated, while autophagy has raised interest. When starved, *Hydra* can survive up to 40 days through autophagy. Starvation periods have a sort of rejuvenation effect on *Hydra* (Schaible et al., 2011), which might be an effect of increased autophagy (Schaible et al., 2014). Starvation does not affect the rate of production of new cells, but the total number of cells decreases. Two parallel processes are observed: in ectodermal cells, autophagy leads to cell survival and rapidly propagates insuring the survival of the animal, while in endodermal cells, (*Kazal1* mediated) autophagy insures the elimination of excess cells by cell death, the rate remaining constant (Chera et al., 2009). In the senescent, cold sensitive strain *Hydra oligactis*, autophagy is deficient in epithelial cells as compared to cold resistant strain of the same species, and might emphasize deficiencies in autophagy in epithelial cells as the main cause of senescence in this strain, while autophagy would be one of the main condition to maintain a continuous rate of cell renewal (Tomczyk et al., 2020).

The role in aging of mitochondrial dysfunction, methylation, transcriptional alteration, modification of histones, chromatin remodeling and the deregulation of nutrient sensing has not been investigated in cnidarians, although all those components are present. Regarding ROS, *Hydra* regulate the expression of mRNAs of superoxide dismutase, glutathione peroxidase and catalases, which are known defenses against ROS (Schaible et al., 2014). Notably, the extreme simplicity of the nuclear envelope architecture in *Hydra* allows for extreme disturbances in the nuclear lamina – *HyLMN*, the only gene coding for lamin in *Hydra*, being expressed in proliferating stem cells only (Klimovich et al., 2018).

#### The Conservation of Decline in the Regenerative Potential of Tissues and Altered Intercellular Communication and the Possible Appearance of Cell Senescence and Inflammation

Stem cells are present in most branches of Eumetazoan, which constitute an important step in their evolution (Bosch, 2009). However, it is a fact without an explanation, why stem cells do not accumulate damage in *Hydra* (Schaible et al., 2017). The three different types of stem cells in *Hydra* illustrate that stem cells must be multifunctional, have different sets of transcription factors, signal transducers and effector genes involved in specific activities related to cell cycle, cell adhesion and cytoskeleton, extracellular matrix, and also have to maintain homeostasis by coordinating one another through ligands-receptors activations mainly via epithelial cells (Hemmrich et al., 2012). The case is different in so-called senescent *Hydra*. Senescent at 10°C (but not at 18°C), *H. oligactis* shows signs of exhaustion of stem cells and disorganization of nervous apical cells and fibers of myoepithelial cells (Yoshida et al., 2006; Tomczyk et al., 2015). In senescent *H. oligactis*, functional, differentiated somatic cells like nematocytes, nerve cells, and actin fibers (which serve as muscle) decline in number and efficiency, which can be interpreted as a form of cell senescence, while the number of stem cells remains constant and the number of germ cells increases during starvation (Yoshida et al., 2006).

A lot of research has focused on the function of FoxO genes in stem cells. Neither choanoflagellates nor plants or fungi, express FoxO (Bridge et al., 2010). FoxO is a family of proteins involved both in the regulation of the insulin pathway and in cell proliferation (Link, 2019). FoxO has been suggested to be involved in longevity through the activation of autophagy, the resistance to oxidative stress and the maintenance of stemness (Martins et al., 2016). FoxO expression is high in all three stem cells lineages in *Hydra*, but low in differentiated cells; *FoxO* silencing increases terminal differentiation and limits growth, while overexpression of *FoxO* induces expression of stemness genes in differentiated nematocytes (Boehm et al., 2012). In *Hydra*, FoxO is expressed mainly in interstitial cells of the ectoderm, that is, the cells that give rise to nematocytes, neurons, secretory cells and gametes, and in reaction to heat shock but not to starvation, which suggests that it might play a protective role for gametes (Bridge et al., 2010). Piwi and Piwi-like genes are expressed mostly in germ line and somatic stem cells, typically in cnidarians, and may constitute an essential part of their regeneration potential (Seipel et al., 2004).

The various species of cnidarians and ctenophores are ideal test cases for the inverse correlation that has often been hypothesized between longevity and level of differentiation or complexity in animals (Petralia et al., 2014). A simple organization without much coordination is easy to maintain, but a more complex one is less easy or impossible to maintain perpetually (Schaible et al., 2017). Intercellular communication is a necessary condition for regeneration in *Hydra* and involves various specific or largely conserved proteins like Wnt and Notch, tyrosine kinases, but not cytokines and growth factors, except for an isomorph of TGF-b, and fibroblast growth factors, all involved in a probably tight balance between renewal and differentiation (Bosch, 2007). Components of the innate immune response (homologs of TLRs and TGF-b) seem to appear as early as in choanoflagellates, possibly with a nutrient sensing function (Richter et al., 2018). Communication between cells and extracellular matrix is essential to regeneration in Hydra (Shimizu et al., 2002). Laminin, a membrane protein that also influences the behavior of attached cells by ligand binding may have appeared in eumetazoans and finds its simplest known expression in cnidarians, where its defects leads to loss of regenerative ability for undetermined reasons (Domogatskaya et al., 2012). Programmed cell death induced by caspase and bcl-2 protein families is found in *Hydra* and play a role in tissue homeostasis (Böttger and Alexandrova, 2007). Bosch has suggested that so-called ‘senescence’ in *Hydra* is the result of an excessive redirection of signaling pathways from differentiation to germline (Bosch, 2009). This may be a very early form of conditional aging through inflammation in some specific circumstances.

The role of the accumulation of senescent cells (Dimri et al., 1995; Liu et al., 2009) and of the increase of systemic inflammation (Franceschi et al., 2000, 2018) during human aging has long been documented.

### Aging in Bilaterians (What Is Common to Bilaterians?)

Bilaterians are characterized by symmetric organization under the control of Homeobox development genes, which regulate cell division, cell death, cell adhesion and cell migration (Pearson et al., 2005). Importantly, some are non-senescent (flatworms like *Schmidtea polychroa*) while other are senescent (*C. elegans*, flatworms like *Macrostomum lignano*), although sometimes with alternative senescent and non-senescent trajectories. Most flatworms that are non-senescent avoid aging through asexual reproduction by fission thanks to germline stem cells expressing *nanos*, controlled shrinkage when starving, and regeneration from body fragments containing neoblasts, that is, pluripotent stem cells expressing *Piwi*, even when neoblasts are injected in an irradiated animal (Petralia et al., 2014). *M. lignano* is a senescent but long-lived flatworm (204 days) with high and non-declining regeneration potential from the head thanks to blastocytes, no rejuvenation and obligatory sexual reproduction: it also shows exceptional resistance to radiation and no cancer (Mouton et al., 2018). Signs of aging are body deformities, grooves in the head, liquid-filled cysts, disintegration of gonads, but also metabolic deregulation (Petralia et al., 2014).

#### Conserved Unicellular Aging in Bilaterians (Degradation of Chaperone-Mediated Protein Folding and Stability, Epigenetic Alterations)

The same kind of protein aggregates as observed in Saccharomyces cerevisiae is observed in the normally aging *C. elegans* (David et al., 2010), that does not have stem cells, and which rate of aging depends on heat shock proteins, while aged individuals also display a high quantity of lipofuscin (Garigan et al., 2002). In this species, the proportion of proteins present in cells of aging animals presented important shifts in balance, reduced in long-lived mutants and enhanced in short-lived ones, a phenomenon that accompanies the long observed accumulation of aggregated proteins (Walther et al., 2015).

The so-called free radical theory of aging, which has emphasized the production of ROS and the accumulation of damage in mitochondria as a major cause of aging in animals (Shigenaga et al., 1994; Balaban et al., 2005), has now become less central in biogerontology, as noted above.

Regarding epigenetic alterations, alterations of chromatin structure through histone modifications have proven to be a major modulator of expression of key genes like *p16*, whose expression increases in senescent cells, and can be curbed notably under the influence of the inactivation of the H3K4 methylase or demethylase, a transmissible factor of longevity in *C. elegans* (O’Sullivan and Karlseder, 2012). Sir2 overexpression is not associated with extended lifespan in all eukaryotes, typically not in *C. elegans* or *D. melanogaster* (Burnett et al., 2011). The expression of sirtuins does not seem to change significantly in association with aging in *Brachionus manjavacas*, a rotifer (Gribble and Mark Welch, 2017).

miRNAs are involved in the control of crucial pathways of aging, like the IGF1-insulin and AMPK pathways, mitochondrial degradation and cell senescence (Smith-Vikos and Slack, 2012). In *C. elegans*, the inactivation of germline cells increases longevity by 60% through the mediation of miRNA *mir-71* in intestinal cells (Boulias and Horvitz, 2012). In *D. melanogaster*, *mir-34* downregulates the expression of the protein E74A, essential in development, during adult life, and the deletion of *miR-34* produces an early-onset catastrophic phenotype of neurodegeneration (Liu et al., 2012).

#### The Underpinnings of Metacellular Aging: Decline in the Regenerative Potential of Tissues, Cell Senescence, Telomere Attrition and Altered Intercellular Communication

Regeneration, extended to the whole body of some planarian or limited to some tissues in narrower clades, declines with age in some bilaterians, not in others, but instead of being an explanation to the process of aging, it is generally considered an effect of the hallmarks of aging (Yun, 2015). It depends on a balance between renewal of stem cells, apoptosis and cell senescence, likely to be regulated by the rate of telomere attrition and important pathways of intercellular communication. Senescent bilaterians seem to have traded off a high proportion of highly active stem cells for more differentiation and organismic complexity.

The absence of stem cells in *C. elegans* may explain that the neutralization of apoptosis has no effect on the lifespan (Garigan et al., 2002). Rotifers have no stem cells either (Snell, 2014). On the contrary, in planarians in general, neoblasts represent 20% of total cells in the organism, with a major and highly effective repair/regeneration function, tightly controlled by proliferation, cell death and autophagy (Aboobaker, 2011). In planarians, the existence of a regulator gene *Smed-p53* that controls proliferation, differentiation and which hyperactivity can even induce exhaustion of stem cells, suggests that a single molecule in the ancestor of bilaterians might have had the functions of antitumoral *p53* family, and self-renewal *p63* and *p73* families (Pearson and Alvarado, 2010). In planarians, *Piwi* genes may be primarily involved in the proper differentiation of neoblasts, and only secondarily in their renewal (Bely and Sikes, 2010).

Defined as the state of arrest of a cell that normally could, but will not divide anymore in a multicellular organism, cell senescence is intimately associated with the other hallmarks of aging, exhaustion of stem cells in particular, in many ways (Dolivo et al., 2016). An important function of cell senescence is to stop cell proliferation, notably, during ontogenesis (Muñoz-Espín et al., 2013; Storer et al., 2013), wound repair, or cancer development. When irradiated, the planarian *Dugesia tigrina* shows a depletion of stem cells and a progressive accumulation of senescent cells over a period of 10 days as measured by a decreased level of H3K27me3 (characteristic of stem cells) and an increased level of traditional markers of cell senescence, such as senescence-associated beta-galactosidase, maybe due to the accumulation of senescent cells in healing wounds (Perrigue et al., 2015).

An inverse correlation between the complexity of the immune system and the capacity to regenerate tissues has been proposed (Peiris et al., 2014). The inflammatory response is considered to originate early in bilaterians, maybe as early as in eumetazoans, depending on how it is defined and what the mediators are supposed to be (Rowley, 1996). Some have suggested that immunity change with age starts with bilaterians, some showing no sign of alteration (planarians) while others do (nematodes) (Ding et al., 2021). However, the picture is no so clear. In *C. elegans*, old individuals tend to accumulate bacteria in the pharynx and intestine, which suggests that they cannot fight infection anymore (Garigan et al., 2002), although it remains unclear what is causing what. The same pathways are chiefly involved in the regulation of innate immunity and in the rate of aging, for example, the DBL-1 pathway, homolog to the mammalian TGF-b pathway (Kurz and Tan, 2004). Several downstream targets of the AMPK pathway are involved in the regulation of inflammation (Salminen et al., 2011), but there is no direct evidence that it is increasing in the nematode. On the other hand, Cathepsin C levels of expression increase in the aging *B. manjavacas*, a typical sign of inflammation in mammals (Gribble and Mark Welch, 2017).

Telomerase activity is high in *Hydra* as it is in Porifera or in planarians that use this mechanism to preserve telomere integrity through a high level of proliferation (Schaible et al., 2014), but this does not seem to be involved in aging in senescent *Hydra*. An important study has cast light on the evolution of telomere attrition in planarians (Tan et al., 2012). The comparison of a sexually and an asexually reproducing strain of the planarian *Schmidtea mediterranea* established that both had insufficient telomerase activity to maintain the length of telomeres in the long run. However, the asexual strain restores its telomere length with each fission whereas the sexual strain restores it during sexual reproduction or during embryogenesis. Interestingly, both strains also have the same regenerative capacity, although its repeated execution leads to severe reduction of telomere length in the sexual strain, where telomerase activity during regeneration is lower, while the increased telomerase activity during regeneration in the asexual strain suffices to renew telomere length. Telomerase seems to already be present in early branching metazoans and even as early as in holozoans (Lai et al., 2017). Recently, it has been suggested that the rate of telomere shortening, not the absolute length of telomeres, is predictive of lifespan in a wide variety of vertebrates (Whittemore et al., 2019).

In humans, the shortening of telomeres with age has first been considered possibly as the main cause of aging, a view that now seems exaggerated. More nuanced views have since been developed, as it was clear that telomere attrition would happen during human aging (Blasco, 2007).

#### Repercussions of Metacellular Aging on Unicellular Aging in Bilaterians: Aggravation of the Loss of Proteostasis and of the Alteration of Nutrient Sensing, Evolution of the Degradation of Nuclear Architecture, and Appearance of Aging Through Accumulation of Mutations

The rate of the degradation of proteostasis has been suggested to depend mainly on the IGF-1 – IIS pathway (Cohen et al., 2006). Other specific proteins are involved in the process, like SKN1, an ortholog of the mammalian Nrf/CNC proteins, which also regulate response to oxidative stress and metabolism (Blackwell et al., 2015). Proteostasis is also degrading in the aging *B. manjavacas*, as several metabolic pathways are also downregulated, including TOR and insulin (Gribble and Mark Welch, 2017).

Importantly, the pathways of nutrient sensing in unicellular organisms has evolved to be sensitive to hormonal cues and build a tissue-specific response in multicellular organisms (Chantranupong et al., 2015). In *C. elegans*, the rate of aging depends on the IGF-1 pathway, and dietary restriction has been shown to have an important effect on lifespan (Garigan et al., 2002). *S. polychroa* is a non-senescent flatworm with periodic and spectacular changes in body size, regeneration and rejuvenation. It does not show any sign of reduced activity with age and stands in opposition to the general law that metabolism is inversely proportional to body mass (Mouton et al., 2011). Lifespan extension in *C. elegans* by manipulation of the IGF-1 – IIS pathway also depends on functional autophagy (Melendez, 2003), but autophagy alone does not seem to have this lifespan extension effect (Hansen et al., 2008). A feedback loop between mitochondrial biogenesis and mitophagy under the control of the SKN-1 transcription factor has been proposed in *C. elegans*, the uncoupling of which accompanies aging, leading to an increased number of dysfunctional mitochondria (Palikaras et al., 2015).

The complexification of the lamina (through the increase in the number of lamin-binding proteins) may be a major cause of the fragility of the nucleus and of cell senescence in bilaterians (Klimovich et al., 2018). Mutations resulting from unrepaired DNA damage become permanent and are primarily driving evolution by natural selection in unicellular organisms, where their accumulation can hardly be considered a cause of aging; in multicellular organisms, the accumulation of mutations in somatic and stem cells only, not in germline cells, is generally considered a cause of aging, including in humans (Vijg, 2021). The conditions for accumulation of mutations to have an effect on aging is that the renewal of somatic or stem cells is not sufficient to prevent mutated somatic cells from having any lasting effect, which occurs in bilaterians.

### Specificities of Aging in Narrower Clades of Bilaterians

All the “hallmarks of aging” have appeared as early as within bilaterians (Table 1). All are therefore likely to be shared across this clade. It would be interesting to establish the disappearance of some of the mechanisms of aging in some narrower clades, as the present review tends to claim that mechanisms of aging can be controlled but are too entrenched in vital mechanisms to disappear altogether. In contrast, evolved mechanisms of anti-aging may disappear. An example is the repression of telomerase production in adult mammals of more than 10kg (Gorbunova et al., 2014). In various species, the respective importance of these mechanisms of aging and mechanisms of anti-aging may lead to more or less pronounced slopes of aging (Jones et al., 2014). Such modulation is likely to explain the exceptional longevity of naked mole-rats (Takasugi et al., 2020), or elephants (Tian et al., 2017).

**TABLE 1:** A recapitulation the various mechanisms of aging that have been shown (or hypothesized) to be involved in aging in the different model organisms mentioned in this review.

	Common clade with bilaterians/humans	Investigated species or taxa	Measurement of aging	Mechanisms of aging involved	References
Unicellular aging	Cellular organism	*Escherichia coli*	Increasing time of reproduction until death	Chaperone-mediated protein folding and stability	Stewart et al., 2005; Ackermann et al., 2007
	Neomura	Archaea	Not investigated	Chaperone-mediated protein folding and stability?	Cavalier-Smith, 2002; Reissmann et al., 2007
				Histone modifications?	Cavalier-Smith, 2002
				Chromatin remodeling?	Ammar et al., 2012
	Eukaryotes	*Chlamydomonas reinhardtii* ciliates *Tetrahymena Pyriformis Euglena gracilis* plants	Chronological lifespan. Replicative lifespan. Clonal aging. Progressive loss of function.	Chaperone-mediated protein folding and stability	Galluzzi et al., 2008
				Histone modifications	Chalker et al., 2013
				Chromatin remodeling	Chalker et al., 2013
				Degradation of proteolytic systems	Pérez-Pérez et al., 2010
				Instable nuclear architecture	Aufderheide, 1986
				Mutations in mitochondrial DNA	Maréchal and Brisson, 2010
				Mitochondrial integrity and biogenesis	Elliott and Bak, 1964; Niyogi, 1999
				ROS	Bender et al., 2008
	Opisthokonts	*Saccharomyces cerevisiae*	Chronological lifespan. Replicative lifespan	Chaperone-mediated protein folding and stability	Zhou et al., 2011
				Histone modifications	Feser et al., 2010
				Chromatin remodeling	Pegoraro et al., 2009
				Degradation of proteolytic systems	Tyler and Johnson, 2018
				Instable nuclear architecture	Pathak et al., 2021
				Mutations in mitochondrial DNA	Longo et al., 2012
				Mitochondrial integrity and biogenesis	Longo et al., 2012
				ROS	
				Deregulated TOR nutrient sensing	Sampaio-Marques et al., 2014
				Deregulated AMPK nutrient sensing	
				Deregulated sirtuin nutrient sensing	
				Deregulated insulin and IGF-1 nutrient sensing	
Metacellular aging	Holozoans	Choanoflagellates	Not investigated	All previous mechanisms likely to be present but not investigated	Richter and King, 2013
				DNA methylation? Transcriptional alterations?	
	Metazoans	Porifera	Chronological lifespan	All previous mechanisms likely to be present but not investigated	Srivastava et al., 2010; Jochum et al., 2012
				Exhaustion of stem cells	Henry and Hart, 2005
	Eumetazoans	*Hydra*	Chronological lifespan. Replicative lifespan.	Chaperone-mediated protein folding and stability, histone modifications, chromatin remodeling, mutations in mitochondrial DNA, mitochondrial integrity and biogenesis, transcriptional alterations, deregulation of nutrient sensing?	Not investigated
				Degradation of proteolytic systems	Schaible et al., 2014; Tomczyk et al., 2020
				ROS	Schaible et al., 2014
				Instable nuclear architecture	Klimovich et al., 2018
				Exhaustion of stem cells	Yoshida et al., 2006; Bridge et al., 2010
				Accumulation of senescent cells	Böttger and Alexandrova, 2007; Bosch, 2009
				Alteration of intercellular communication	Bosch, 2007
	Bilaterians	*Caenorhabditis elegans Schmidtea polychroa Schmidtea mediterranea Brachionus manjavacas Drosophila melanogaster*	Chronological lifespan	Chaperone-mediated protein folding and stability and degradation of proteolytic systems	Garigan et al., 2002; David et al., 2010
				Histone modifications	O’Sullivan and Karlseder, 2012
				Chromatin remodeling	
				Instable nuclear architecture	Klimovich et al., 2018
				Mutations in mitochondrial DNA	Palikaras et al., 2015
				Mitochondrial integrity and biogenesis	
				ROS	Balaban et al., 2005
				TOR nutrient sensing	Smith-Vikos and Slack, 2012; Chantranupong et al., 2015
				AMPK nutrient sensing	
				Sirtuin nutrient sensing	
				Insulin and IGF-1 nutrient sensing	
				DNA methylation Transcriptional alterations	Smith-Vikos and Slack, 2012; Greer et al., 2015
				Exhaustion of stem cells	Aboobaker, 2011
				Accumulation of senescent cells	Smith-Vikos and Slack, 2012; Dolivo et al., 2016
				Alteration of intercellular communication	Kurz and Tan, 2004; Ding et al., 2021
				Telomere shortening	Tan et al., 2012
				Accumulation of nuclear DNA mutations	Vijg, 2021

A further characterization of human aging relies on yet more specific hallmarks of aging, specific to humans or shared with other species. An example is the progressive reduction of the repertoire of T cells, one of the components of human immunosenescence (Weiskopf et al., 2009) that may strike all living organisms with an adaptive immune system, namely, gnathostomes (Cooper and Alder, 2006).

It is also important to take into account the specificities of the aging of some organs or systems. The vascular system is common ancestor to protostomes and deuterostomes, with the endothelium appearing later on in vertebrates (Monahan-Earley et al., 2013); but it is unclear when the aging of the endothelium has appeared in evolution. More generally, the specificities of aging in some species is likely to explain some striking variations in the risk of certain age-related diseases, as is the case with cancer (Schiffman and Breen, 2015).

## Discussion

Bilaterian, and thereby human aging consists in an evolved, multilayered mechanism (represented in Figure 3). A new layer appears when a mechanism A is responsible for a progressively degrading function, i.e., an aging mechanism B. Mechanism A then becomes a mechanism of aging. All the known layers of aging are present as widely as in bilaterians.

The first layer of aging is the accumulation of unfolded or unstable proteins. As it appears as early as in unicellular organisms, it is universal. In other terms, no species is devoid of at least one mechanism of aging, although in some, its effects are efficiently countered by mechanisms of anti-aging. The first mechanism of anti-aging is disposal by cell division.

The second layer of aging is epigenetic alterations under the form of chromatin remodeling and histone modifications. It has appeared with the evolution of a more sophisticated support for DNA and does not seem to be causally related to the first layer. It concerns all archaea and eukaryotes.

The third layer of aging contains mitochondrial dysfunction, more specifically, ROS damage and the progressive degradation of mitochondrial integrity and biogenesis, damage to mtDNA and damage to the nuclear architecture, and finally the progressive degradation of proteolytic systems. The appearance of these mechanisms of aging is apparently unrelated to the existence of the previous ones. Yet, interactions are likely: the generation of ROS may increase the number of misshaped proteins, the loss of mitochondrial integrity may increase the generation of ROS. The mechanisms of the third layer result from the appearance of the characteristics of eukaryotic life, the existence of a nucleus, of mitochondria (and chloroplasts), and the appearance of autophagy. All eukaryotes share the mechanisms of this third layer – except those who have possibly lost one of its components (Karnkowska et al., 2016).

The fourth layer of aging contains all the mechanisms grouped under the label of ‘nutrient sensing’: sirtuins and the TOR, AMPK and Insulin – IGF-1 pathways. These mechanisms also appeared independently from mechanisms of the first three layers. However, the level of interactions increases dramatically with this layer, which may be interpreted as a mechanism focused on the management of the available energy sources that happens to control many of the mechanisms of aging of the first three layers (directly with the regulation of autophagy or mitochondrial activity, indirectly through the double role of sirtuins in the regulation of this mechanism and in genomic maintenance), and thereby modulate the rate of aging. These mechanisms characterize opisthokonts, but not all eukaryotes, as their components do not seem to be involved in aging in bikonts, although most of them are present.

These first four layers of aging together constitute the hallmarks of *unicellular* aging. Unicellular organisms contain some or all of them and most multicellular opisthokonts still contain all of them. In unicellular organisms, the problem of unicellular aging is mainly solved through reproduction, sexual or clonal, which resets the aging clock for at least one of the two cells that result from division.

The fifth layer of aging contains DNA methylation and transcriptional alterations. In general, these epigenetic mechanisms, appeared early during the evolution of unicellular organism, have the effect of modulating the expression of genes in a cell, which is necessary to the coordination of individual cells in multicellular life. There is evidence that they are involved as mechanisms of aging in metazoans but it is plausible that they are involved in the aging of a colony in holozoans.

The sixth layer of aging is the decline in the regenerative potential of tissues. It appears with the distinction between stem cells and somatic cells in metazoans. Importantly, this duality of cells is an elegant multicellular solution to the problems of unicellular aging, as long as damaged somatic cells can be renewed, and as the renewal of stem cells can outpace the accumulation of damage as efficiently as prokaryotes get rid of accumulated protein aggregates by sequestrating them into one lineage. When the renewal of cells is insufficient, multicellular organisms age.

The seventh layer of aging contains both inflammation and the accumulation of senescent cells. The mechanisms of aging in this layer are likely to be strongly dependent on the existence of a lower rate of renewal of the cells in a multicellular organism, although they probably originate in some of the specificities of eumetazoans. Inflammation, cell senescence and the decline in the regenerative potential of tissues together form the engine of aging in most senescent multicellular organisms.

The eighth and last layer of aging contains the accumulation of mutations in nuclear DNA, telomere attrition and alterations of other forms of intercellular communications as those involved in inflammation. These mechanisms of aging do not depend on the appearance of new entities with bilaterians, but on the considerable complexification of intercellular communication and mutual dependency that appears at this stage, under the constraint of the existence of a complex organization.

The last four layers of aging together constitute the hallmarks of *metacellular aging*, that is, the aging of the cells of the organism that happens in multicellular life only. Metacellular aging is the problem of aging left unsolved by evolution in many metazoans. It basically consists in the failure to control the effects of unicellular aging, so that they progressively affect the whole multicellular organism, which eventually dies. Importantly, multicellular organisms may also control unicellular aging by other mechanisms than the renewal of cells, possibly, the regulation of unicellular aging. For instance, aging mammals show significantly less protein aggregates than *C. elegans*, pointing toward more a more efficient protein turn-over (Walther et al., 2015).

In the end, although the multilayer view of aging casts considerable light on the general process of aging, there are three important limitations, that all stem from the essentially ‘basic cell biology’ approach to aging taken in López-Otín et al. (2013). The first is that it ignores potentially important non-cellular factors of multicellular aging, like the continuous remodeling, and progressive structural degradation, of the extracellular matrix. The second is that it does not describe how variations of the general mechanism of aging explain the huge variety of the rate of aging among bilaterians. The third is that the importance, and maybe even the implication of some mechanisms of aging may depend on environmental factors, as shown in the example of *Furcifer labordi* (Cohen et al., 2020).

The present review has not taken a comparative view of aging in different branches. An important question is whether metazoans and plants justify different definitions of aging because of their different patterns of metacellular aging. Basically, plants have a much more modular structure than metazoans, close to that of colonial organisms. Aging seems to be a more local phenomenon touching the phytomere (basic functional unit of the plant) rather than the plant itself, and while aging refers to the whole lifecycle of the plant, senescence (of the leaf) is a necessary phase of nutrient remobilization (Avila-Ospina et al., 2014) that may or may not lead to death. Moreover, senescence and death are related to competition between the sink (net importer of nutrients) and the source (providing precursors for sink metabolism). Several molecular mechanisms of aging may be involved in both metazoans and plants – epigenetic changes, loss of proteostasis (autophagy), nutrient sensing (TOR), intercellular communication (e.g., hormones and cytokinins in plants), telomere shortening – but they do not seem to have the same importance in both kingdoms, and some mechanisms, like accumulation of mutations, are unlikely to play a role in aging in plants (Thomas, 2013), but may instead play a role in the evolution and adaptation of the species to pest (Plomion et al., 2018). Both in animals and plants, the role of the production of ROS in mitochondria and in chloroplasts (in plants) has recently been reevaluated from a simple toxic byproduct to a possible regulator of various processes (Singh et al., 2016).

This multilayered view of aging is subject to potential revisions. First, it relies on a lacunary literature on aging in many clades and depends on the choice of model organisms (Box 1). In particular, the investigation of replicative lifespan in archaea should cast some light on the transition to eukaryotic aging, while the investigation of the various mechanisms that limit the lifespan of the colony in holozoan and the investigation of conditional senescence in cnidarians and ctenophores should explain more on the onset of metacellular aging. Second, I have made some explicit hypotheses on the appearance of aging though DNA methylation, transcriptional alterations and decline in the regenerative potential of tissues for lack of more specific studies in the literature. I have also proposed surprising hypotheses that many will find questionable – such as the late appearance of aging through nuclear DNA mutations.

**Box 1. Is *Caenorhabditis elegans* a good model of bilaterian aging?**

*Caenorhabditis elegans* is one of the standard models in aging research. Many mechanisms of aging have been investigated, and aging-retarding drugs tested, on this model organism. Mechanisms include: chaperone-mediated protein folding and stability (Walther et al., 2015), histone modifications (Yi and Kim, 2020), chromatin remodeling (Greer et al., 2010), degradation of proteolytic systems (Hansen et al., 2008), nuclear architecture (Romero-Bueno et al., 2019), of mtDNA (Trifunovic and Larsson, 2008), of mitochondrial integrity and biogenesis (Palikaras et al., 2015), ROS-inflicted damage (Finkel and Holbrook, 2000), alteration of IIS-IGF1 nutrient sensing (Lin et al., 1997), Sirtuins nutrient sensing (Baur and Sinclair, 2006), AMPK nutrient sensing (Greer et al., 2007), TOR nutrient sensing (Hansen et al., 2008), transcriptional alterations (Smith-Vikos and Slack, 2012), DNA methylation (Greer et al., 2015), inflammation (Salminen et al., 2008), alterations in intercellular communication (Kurz and Tan, 2004; Ermolaeva and Schumacher, 2014).

However, *Caenorhabditis elegans* contains postmitotic cells only. For this reason, most characteristics of metacellular aging are, in this organism, different from most bilaterian organisms, including decline in the regenerative potential of tissues, accumulation of senescent cells, telomere attrition, and accumulation of mutations in nuclear DNA. This explains the main limit of extrapolation from aging in this worm to aging in most bilaterians, including mammals. If metacellular aging is indeed the core of aging in senescent multicellular species, then the nematode is ill-suited to investigate its mechanisms. On the other hand, it is a paradoxically good model to study unicellular aging in a multicellular organism without most of the layers of metacellular aging.

## Conclusion

This paper has proposed a multilayered evolutionary view on aging (MEVA). This view shows more concretely than the ETA how aging may have evolved until bilaterians. It also constitutes the first necessary step to build a comprehensive view of how aging works. In particular, it suggests a dichotomy between layers of unicellular aging and layers of metacellular aging.

This dichotomy in turn sketches the potential impact and limits of various anti-aging interventions. To put it in a nutshell, some interventions target layers of unicellular aging, like the TOR pathway, mitochondria or DNA damage, while other target layers of multicellular aging, like senolytics. Targeting unicellular aging may slow down the rate of accumulation of dysfunctional cells with time while targeting multicellular aging may stimulate the replacement of these accumulating dysfunctional cells. If non-senescent animal species massively illustrate the latter strategy rather than the former, as it seems to be the case, it is a clear sign of its superiority. Moreover, slowing down the accumulation of damage to somatic cells does not imply that their replacement will be easy. On the other hand, it is both necessary that stem cells are to some extent protected from unicellular aging, and unclear how they can avoid the accumulation of damage apart from a very high rate of proliferation, in a way that is very much reminiscent of how prokaryotes avoid limited RLS. To flesh out a more specific hypothesis, it would probably be of interest to systematically review the effects of the various anti-aging compounds that have been tested on various models, not only on the lifespan or on senescence, but on the various mechanisms of aging and anti-aging.

It is also indispensable to systematically review the various modulations of these mechanisms that can be observed in various bilaterian species, not necessarily to imitate them for interventions on human aging, but also to understand what can and what cannot work to achieve this practical goal.

In the end, genetic variations relevant for human aging combined with the diversity of environments will also cast light on how these mechanisms interact.

## Author Contributions

The author confirms being the sole contributor of this work and has approved it for publication.

## Conflict of Interest

The author declares that the research was conducted in the absence of any commercial or financial relationships that could be construed as a potential conflict of interest.

## Publisher’s Note

All claims expressed in this article are solely those of the authors and do not necessarily represent those of their affiliated organizations, or those of the publisher, the editors and the reviewers. Any product that may be evaluated in this article, or claim that may be made by its manufacturer, is not guaranteed or endorsed by the publisher.
